# Catheter ablation for atrial fibrillation in left ventricular assist device: A case report

**DOI:** 10.1097/MD.0000000000026308

**Published:** 2021-06-25

**Authors:** Yu Jin Chung, Jin-Oh Choi, Kyoung-Min Park

**Affiliations:** Division of Cardiology, Department of Medicine, Heart Vascular and Stroke Institute, Samsung Medical Center, Sungkyunkwan University School of Medicine, Seoul, South Korea.

**Keywords:** atrial arrhythmias, atrial fibrillation, heart failure, left ventricular assist device, radiofrequency catheter ablation

## Abstract

**Introduction:**

Mechanical circulatory support such as the left ventricular assist device (LVAD) has become widely implemented in the treatment of end-stage heart failure, whether as bridge-to-transplant or as destination therapy. The hemodynamic effects of arrhythmia on LVADs and its management are significant in determining the long-term outcome of these patients. Both atrial arrhythmia and ventricular arrhythmia are commonly seen after implantation of the device. There are no strict guidelines, however, on the need for intensive management of arrhythmias in LVAD. In this case report, we present a patient with new onset atrial fibrillation after LVAD implantation which leads to acute decompensating heart failure. The patient was treated with catheter ablation. The intervention demonstrated positive outcomes for this patient.

**Patient concerns:**

The patient was a Korean male, who presented with dyspnea, fatigue and generalized edema after persistent atrial fibrillation precipitated by implantation of the left ventricular assist device.

**Diagnosis:**

The patient was diagnosed with acute decompensating heart failure that was aggravated by recurrent atrial arrhythmia.

**Intervention:**

We attempted to relieve symptoms of right ventricular dysfunction by method of strict rhythm control in this patient. The patient underwent radiofrequency catheter ablation for recurrent atrial fibrillation.

**Outcome:**

The patient showed improved clinical symptoms, BNP levels, and echocardiogram parameters immediately after the procedure as well as during long term outpatient follow up.

**Conclusion:**

In this case report, we present the first successful case in Korea of atrial fibrillation in LVAD treated with catheter ablation. This case suggests setting catheter ablation as a routine first-line treatment for atrial arrhythmia in LVAD patients, especially when the arrhythmia predisposes the patient at risk for decompensating heart failure.

## 1 Introduction

New-onset atrial fibrillation (AF) is prevalent among patients with left ventricular assist device (LVAD) implantation.^[1]^ Although the clinical impact of atrial arrhythmias (AA) on LVAD recipients is not entirely clear, sustained AAs predispose patients to a risk of right heart failure.^[2]^ Several studies have shown that AAs in LVAD support are associated with increased readmission and mortality rates.^[3,4]^ Inadequate optimization of LVAD support precipitates right ventricular failure, as septal deviation toward the inflow cannula leads to right ventricle (RV) dilatation and wall stress. Due to the lack of atrial support to ventricular filling, AAs can cause atrioventricular dyssynchrony, which can adversely impact RV systolic and diastolic function.^[4]^ Because of the critical nature of RV function in LVAD patients, controlling AAs may decrease the risk of RV failure in those requiring LVAD support. There is no clear consensus on the appropriate treatment of arrhythmias in LVAD, and there are few reports on the success rates of catheter ablation in LVAD. Ventricular arrhythmias (VA) in LVAD have been more thoroughly investigated, and case reports have shown successful ablation of VAs.^[5]^ The first case of catheter ablation in atrial arrhythmias was reported in 2010.^[6]^ Maury et al^[6]^ performed radiofrequency catheter ablation (RFCA) in a patient with a HeartMate II LVAD without any known complications. Hottigoudar et al^[7]^ presented the largest series of AF treated with catheter ablation in LVAD patients, and showed significant improvement in both symptoms and follow-up ECGs. We report the first case in Korea of a patient with post-LVAD AF treated with RFCA.

## 2 Case report

**Figure 1:**
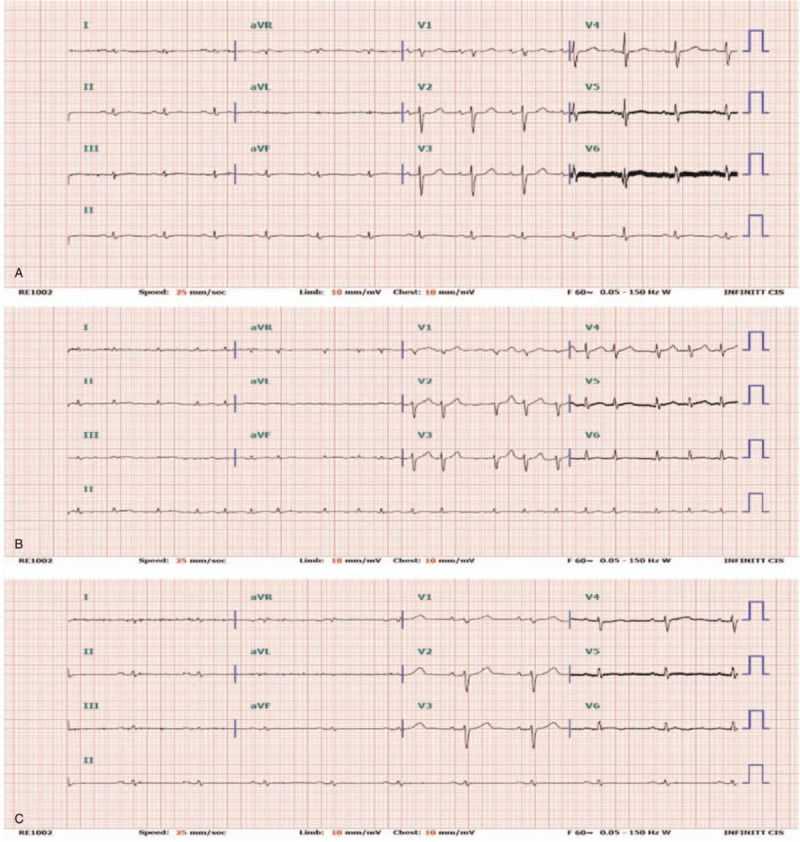
12-lead ECGs. (A) Sinus rhythm, post-LVAD (B) Persistent atrial fibrillation, pre-RFCA (C) Sinus rhythm, post-RFCA. ECG = electrocardiogram, LVAD = left ventricular assist device, RFCA = radiofrequency catheter ablation.

A 77-year-old man with a history of paroxysmal AF and dilated cardiomyopathy with severe medically refractory heart failure underwent LVAD implantation. ECGs showed sinus rhythm after LVAD implantation (Fig. 1a).

Two years after LVAD insertion, however, the patient experienced several episodes of AF with rapid ventricular response. These episodes of arrhythmia caused a decrease in LVAD flow, resulting in symptoms of right heart failure. He complained of dyspnea, generalized edema and fatigue. Echocardiogram showed a decreased left ventricular systolic function of 25% and increased tricuspid valve regurgitation with an enlarged RV cavity. RVSP was significantly increased from 34 to 65.4 mmHg. Antiarrhythmic drugs including amiodarone and digoxin were used, but sinus conversion was not achieved. Sinus rhythm was restored with DC cardioversion. With successful rhythm and rate control, the patient showed improvement of symptoms. However, sinus conversion was temporary and his persistent AF refractory to medical treatment aggravated symptoms of right heart failure (Fig. 1b). Follow-up echocardiogram showed a decreased ejection fraction and increased RVSP. To maintain sinus rhythm, RFCA was performed (Fig. 3). Immediate postoperative ECGs showed successful sinus conversion and clinical improvement (Fig. 1c).

**Figure 2:**
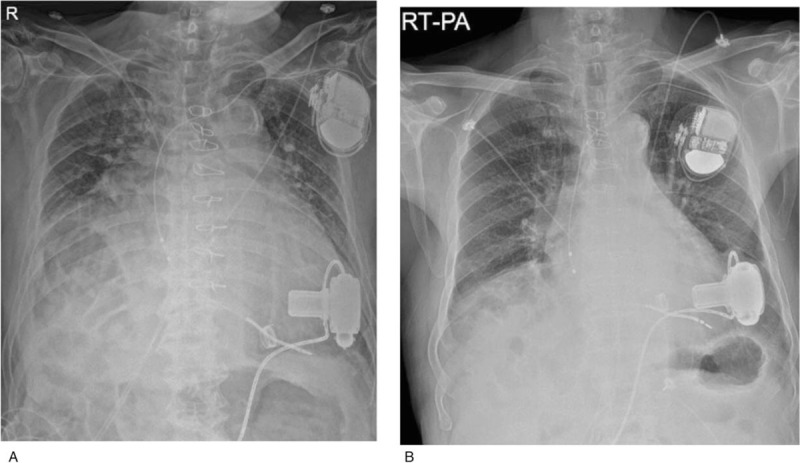
Chest radiography. (A) pre-RFCA shows bilateral haziness and pulmonary edema (B) Image before discharge after successful RFCA shows improved pulmonary edema and lung condition. RFCA = radiofrequency catheter ablation.

Two days after the procedure, the patient presented with fever and dyspnea. His chest X-ray showed pulmonary edema with combined pneumonia (Fig. 2a). With antibiotics, inotropic medications, and optimization of LVAD pump speed, the patient had relief of clinical symptoms. He was discharged from the hospital with improved chest x-ray and normal sinus rhythm on ECG (Fig. 2b).

**Figure 3:**
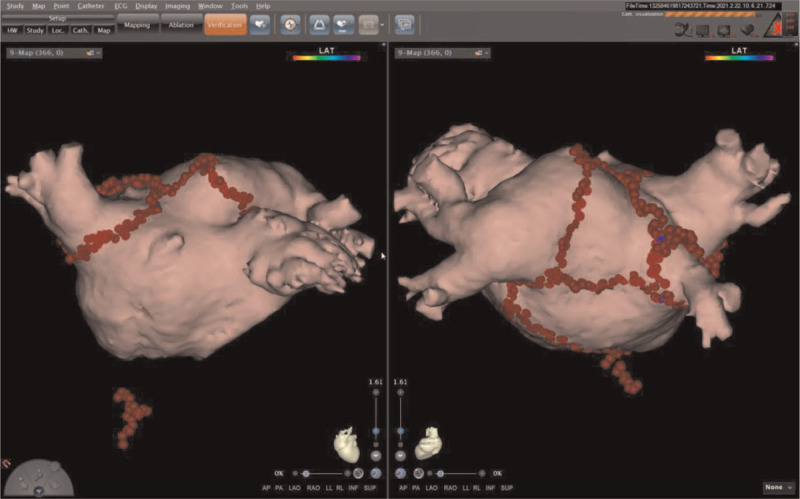
3-Dimensional reconstruction of radiofrequency catheter ablation of atrial fibrillation.

After eight months of follow-up, ECGs consistently showed normal sinus rhythm. Serum BNP levels fell from 9665 to 417pg/mL. The patient has remained free of symptoms of low cardiac output.

## 3 Discussion

LVAD patients commonly experience arrhythmias, which can modify hemodynamics in ways that may have a clinical impact on the outcome and prognosis of heart failure.^[2]^ However, there is no consensus on the best practices for medical management of these new-onset arrhythmias, or on the optimal timing for procedural interventions. We report this case of a patient who redeveloped atrial arrhythmia two years after LVAD implantation and experienced recurrent episodes of atrial fibrillation, leading to right heart failure. Atrial arrhythmia was the main precipitating factor contributing to the patient's right heart failure in this case. We focused on rhythm control as the key strategy for treatment. Six months after the procedure, the patient has shown improvement of low cardiac output symptoms and LV function on follow-up echocardiogram.

Although the general management of AAs is well-reported for non-LVAD heart failure patients, managing LVAD patients seems to be entirely different.^[8]^ The effects of rate control in LVAD patients are unproven, but the consensus is that beta blockers are usually used in LVAD patients who have AF.^[4]^ The combination of beta blockers and digoxin is typically approved for the management of AF.^[9]^ The efficacy of rhythm control medication is more limited. Several reports have used amiodarone or dofetilide to control AF in LVAD patients.^[9]^ In hemodynamically unstable AF, other measures should be taken with caution, but there is a paucity of data available, and RFCA needs to be further studied.^[3,10,11]^ Our case is significant in that it reports a successful case of RFCA in controlling medically refractory AF. Furthermore, we should consider applying RFCA as early first-line treatment in LVAD patients for both symptomatic and clinical improvement, due to side effects of the rate and rhythm controlling drugs.^[3,8–10]^

The major limitation to this study is the practicality in identifying the timing of intervention. Atrial arrhythmia commonly occurs concurrently in patients with heart failure and oftentimes antiarrhythmic drugs are used as first-line treatment. Recognizing the significance of early intervention with catheter ablation will need to be investigated in more cases. These studies will need to be mostly studied in large centers that are experienced in management of LVAD as well as catheter ablation.

## 4 Conclusion

This is the first case reported in Korea of a patient with post-LVAD AF which was treated with RFCA. This case demonstrates the clinical implications of new-onset AF in LVAD, with regard to its association with RV failure and hemodynamic deterioration as well as its management.

Long-term ECG monitoring and optimal timing of treatment for AF in LVAD requires further discussion.

## Author contributions

**Conceptualization:** Jin-Oh Choi.

**Supervision:** Kyoung-Min Park.

**Writing – original draft:** Yu Jin Chung.

**Writing – review & editing:** Kyoung-Min Park, Yu Jin Chung.
